# Metabolomics, Standards, and Metabolic Modeling for Synthetic Biology in Plants

**DOI:** 10.3389/fbioe.2015.00167

**Published:** 2015-10-21

**Authors:** Camilla Beate Hill, Tobias Czauderna, Matthias Klapperstück, Ute Roessner, Falk Schreiber

**Affiliations:** ^1^School of BioSciences, University of Melbourne, Parkville, VIC, Australia; ^2^Faculty of Information Technology, Monash University, Clayton, VIC, Australia; ^3^Institute of Computer Science, Martin Luther University Halle-Wittenberg, Halle, Germany

**Keywords:** metabolic engineering, synthetic biology, metabolic modeling, systems biology, metabolomics

## Abstract

Life on earth depends on dynamic chemical transformations that enable cellular functions, including electron transfer reactions, as well as synthesis and degradation of biomolecules. Biochemical reactions are coordinated in metabolic pathways that interact in a complex way to allow adequate regulation. Biotechnology, food, biofuel, agricultural, and pharmaceutical industries are highly interested in metabolic engineering as an enabling technology of synthetic biology to exploit cells for the controlled production of metabolites of interest. These approaches have only recently been extended to plants due to their greater metabolic complexity (such as primary and secondary metabolism) and highly compartmentalized cellular structures and functions (including plant-specific organelles) compared with bacteria and other microorganisms. Technological advances in analytical instrumentation in combination with advances in data analysis and modeling have opened up new approaches to engineer plant metabolic pathways and allow the impact of modifications to be predicted more accurately. In this article, we review challenges in the integration and analysis of large-scale metabolic data, present an overview of current bioinformatics methods for the modeling and visualization of metabolic networks, and discuss approaches for interfacing bioinformatics approaches with metabolic models of cellular processes and flux distributions in order to predict phenotypes derived from specific genetic modifications or subjected to different environmental conditions.

## Introduction

One of the greatest challenges for scientists is to understand the genetics, physiology, and biochemistry of plants and the interaction of genes with the environment in order to provide strategies to manipulate these processes to improve plant growth and performance and to prevent diseases. There have been immense advances in the past decades allowing scientists to sequence genomes of organisms addressing many questions, such as how the genome determines a plant’s response to any environmental stimuli. Simultaneously, the development of analytical technologies allows us to take a comprehensive and unbiased glance at the gene products, such as gene transcripts (mRNA), proteins, lipids, and metabolites. The post-genomics era was born with the establishment of transcriptomics, proteomics, lipidomics, and metabolomics, each with their associated computational advances providing the path for data analysis, visualization, and integration to establish the relationships between the genome and gene products under certain conditions.

Metabolites are synthesized by enzyme-catalyzed reactions in any living cell. They are important for the maintenance and survival of cells, most importantly for energy storage and provision, and they also contribute to building and maintaining the cell’s structural components. Metabolites and their functionalities are indispensable in the interaction of a cell with the environment, and it has been argued that the metabolome of any biological system represents the final “read-out” of the expression of many genes in that system in a particular situation, and reflecting gene × environment relationships (Hill et al., 2014).

In comparison to transcripts or proteins, metabolites have a vast range of different chemical structures with an astonishing array of different functional groups that lead to differences in their physical and chemical properties, such as solubility, reactivity, stability, and polarity (Trethewey, 2004). This sheer diversity presents challenges to assay these compounds in a multiparallel fashion. Firstly, a number of different solvent extraction procedures need to be utilized to extract metabolites efficiently from any given plant tissue. In addition, no single analytical approach is capable of detecting and quantifying such chemical diversity; therefore, a range of different approaches (more detail below) has to be employed to analyze as many metabolites as possible. Today, metabolomics is considered as the science combining modern and sophisticated analytical instrumentation for metabolite detection and quantification with appropriate computational and statistical approaches to extract, mine, and interpret metabolomics data.

Metabolomics is now also becoming an important tool for biotechnological and metabolic engineering approaches, which aim to manipulate biochemical pathways to enhance the accumulation of compounds of interest (Dromms and Styczynski, 2012). Since metabolomics can provide a more complete picture of the biological system studied, it has been argued that it can be applied to identify metabolite markers that indicate a particular phenotype [e.g., level of target compound(s)] to allow the assessment of the successes of engineering steps to provide further guidance for future engineering strategies (Beckles and Roessner, 2011). Historically, metabolic engineers have used the analysis of the levels of the target compound(s) and potentially a few closely related metabolites to define metabolic engineering strategies. However, the potential metabolomics offers, which measures hundreds of metabolites rather than just a few, has only been rarely explored in metabolic engineering approaches, particularly in plants (Rios-Estepa and Lange, 2007; Fernie and Morgan, 2013). The unbiased and broad approach of metabolomics helps to assess how plants maintain energy, carbon, and nutrient resources and provides information how these resources may potentially be redirected into the synthesis of the desired metabolites, therefore allowing smart engineering strategies to be developed.

In the following, we review current metabolomics technologies, information resources for metabolomics, as well as computational analysis and modeling approaches with a focus on plant related research.

## Tools and Technologies to Study Plant Metabolism

### Analytical Technologies

The plant metabolome, compared to the metabolome of other organisms, is represented by a particularly vast variety of chemical structures with an enormous diversity of chemical and physical properties (Villas-Boas et al., 2007). In the past decade, researchers have developed and validated a number of complementary analytical approaches to extract, separate, detect, and quantify this diversity. Different solvent extraction procedures may need to be employed to cover the range of polarity of metabolites (Dias et al., 2012). However, most routine metabolite extractions are based on a methanol/water/chloroform biphasic extraction, which captures a large complement of the plant metabolome.

Once metabolites are extracted, the complex mixtures need to be separated allowing individual detection and quantification of compounds. The polarity of metabolites also influences the choice of the separation approach. In liquid chromatography (LC)-based separations, researchers now commonly use two types of separation chemistries, such as C18 reverse phase, which separates the more hydrophobic complement of a metabolite extract, such as many secondary metabolites and lipids, and hydrophilic interaction chromatography, which is better suited for the polar metabolites (Callahan et al., 2009; Hill and Roessner, 2015). In addition, many metabolites are either positively or negatively charged molecules, therefore molecules need to be ionized using both positive and negative ionization mode (Beckles and Roessner, 2011; Hill et al., 2014). There are a number of different ionization techniques available, such as electrospray ionization or atmospheric pressure ionization, again each better suited for a particular subclass of metabolites. However, the most common technique used in LC-MS-based metabolomics is electrospray ionization, which allows reliable ionization of thousands of compounds (Hill et al., 2014).

An alternative separation technique is gas chromatography (GC), which is known for its superior separation power and reproducibility (Dias et al., 2015). The Metabolomics Standards Initiative (MSI; Fiehn et al., 2007) has developed minimal reporting standards for metabolomics data, and strategies to further enhance reproducibility, experimental and data standardization are continuously developed (Allwood et al., 2009). However, GC requires compounds to be volatile to be amenable for analysis. Most metabolites are not volatile and therefore require chemical derivatization to make them volatile. This limits the utility of GC-based separation in metabolomics applications; however, GC coupled to MS still remains the “work horse” in metabolomics due to its reproducibility and its ease of use (Hill and Roessner, 2013).

Mass spectrometry (MS) is the most commonly used detector in metabolomics approaches. The power of MS is that it can distinguish the size of the ionized molecule (by determining the mass to charge ratio of each detected ion) and allows the determination of the number of individual ions detected. The mass to charge ratio determined by MS is used for compound identification and the number of ions detected can be related back to initial concentration of the molecule in the metabolite extract. Another advantage of MS is that it can fragment an ion once (MS/MS) or multiple times (MS^n^), a feature used for structural elucidation of unknown compounds. MS also has excellent capabilities to detect and distinguish isotopic patterns of each ion under analysis. This is particularly important for stable isotope labeling experiments used for metabolic flux analysis. MS analysis is able to determine enrichments in stable isotope labels in all the analyzed compounds and therefore allows determining metabolic fluxes through particular pathways. Most commonly ^13^C labeled metabolic precursors are used, and the distribution and enrichment of the label across pathways of interest are quantified (O’Grady et al., 2012; Chokkathukalam et al., 2014). ^13^C-labeling patterns can also be detected by nuclear magnetic resonance spectroscopy (NMR), which also represents an important platform for untargeted, non-destructive metabolite profiling (Eisenreich and Bacher, 2007).

### Metabolomics Data Analysis and Visualization

Metabolic data give us a snapshot of the current state of an organism (Hill and Roessner, 2013). It represents the outcome of a preceding gene expression profile, which influences the activity of pathways, transport processes, as well as production and consumption of metabolites. The resulting metabolic profile can be used to classify organisms, for example, by genotype or treatment. Furthermore, these profiles enable comparative analysis between selected treatments or genotypes (Hill et al., 2013a,b, 2015) and to obtain information about metabolites with most or least changes as a result of changes in the gene expression profile.

There are two general methods to analyze metabolic data, which can also be combined. The first, analytical method uses commonly known statistics and clustering algorithms, the second method implies the use of networks to visualize spatial and temporal properties of the data. Performing data statistics in the scope of networks for visualization purposes represents the combination of both methods.

Scientists can choose from a broad repository of statistical methods with respect to the objective at hand. Methods such as frequency distribution, analysis of variance (ANOVA), min–max, and Pearson’s and Spearman-rank correlation are examples for univariate data analysis. Principal component analysis (PCA), partial least squares regression (PLS), and multivariate analysis of variance (MANOVA) are commonly used for multivariate data analysis. Self-organizing maps (SOM), support vector machines (SVM), and k-means are popular methods for cluster analysis. Generated results can be visualized using different kinds of diagrams such as plots, histograms, cluster diagrams, and heat maps.

To perform the above-mentioned analytical methods, several software tools can be used. There is a distinction between low-level tools to provide the actual set of algorithms, which are in turn used by high-level tools to provide user-friendly application of those algorithms. Table 1 shows a selection of frequently used software tools.

**Table 1:** **Software tools for metabolomics data analysis**.

Name	Reference	URL
**HIGH LEVEL**		
MetaboAnalyst	Xia et al. (2015)	http://www.metaboanalyst.ca/
XCMS	Gowda et al. (2014)	https://xcmsonline.scripps.edu/
MetATT (Metabolomics tool for analyzing two-factor and time-series data)	Xia et al. (2011)	http://metatt.metabolomics.ca/MetATT/
metaP-Server	Kastenmüller et al. (2011)	http://metap.helmholtz-muenchen.de/metap2/
**LOW LEVEL**		
Matlab (PLS toolbox, msalign, etc.)	MathWorks (2012)	http://ch.mathworks.com/products/matlab/
R-packages (AmsRPM, apLCMS, metabolomics, muma, etc.)	R Development Core Team (2008), Kirchner et al. (2007), Yu et al. (2009), Gaude et al. (2013)	http://www.r-project.org/

Graphical representations of metabolic pathways and networks have been used for long time to represent knowledge about metabolic processes, and with the availability of pathways in databases, several tools have been developed to visualize metabolic data in the context of networks. These tools mostly support tasks such as data mapping and network analysis but often also try to help with layout and exploration of data. The latter touches the field of visual analytics for metabolic information (Kerren and Schreiber, 2012, 2014). Table 2 shows a selection of tools supporting visualization of metabolic data as diagrams or heat maps in the context of biological networks.

**Table 2:** **Network visualization software tools that support metabolic data**.

Name	Reference	URL
MetScape Plugin for Cytoscape	Karnovsky et al. (2012)	http://metscape.ncibi.org/
MetaMapp (MS data in context of metabolic networks using Cytoscape)	Barupal et al. (2012)	http://metamapp.fiehnlab.ucdavis.edu
MAVEN (LC-MS)	Clasquin et al. (2012)	http://maven.princeton.edu
VANTED	Rohn et al. (2012)	http://www.vanted.org
Pathomx	Fitzpatrick et al. (2014)	http://pathomx.org/

As a result of applying common procedures of extracting and measuring metabolic abundance and concentration, usually metabolomic data possesses no spatial information. With the advent of modern methods such as imaging MS (Kaspar et al., 2011; Miura et al., 2012), additional spatial information can be gathered and displayed using 2D (Rohn et al., 2011) or 3D immersive techniques (Sommer et al., 2011).

**Figure 1:**
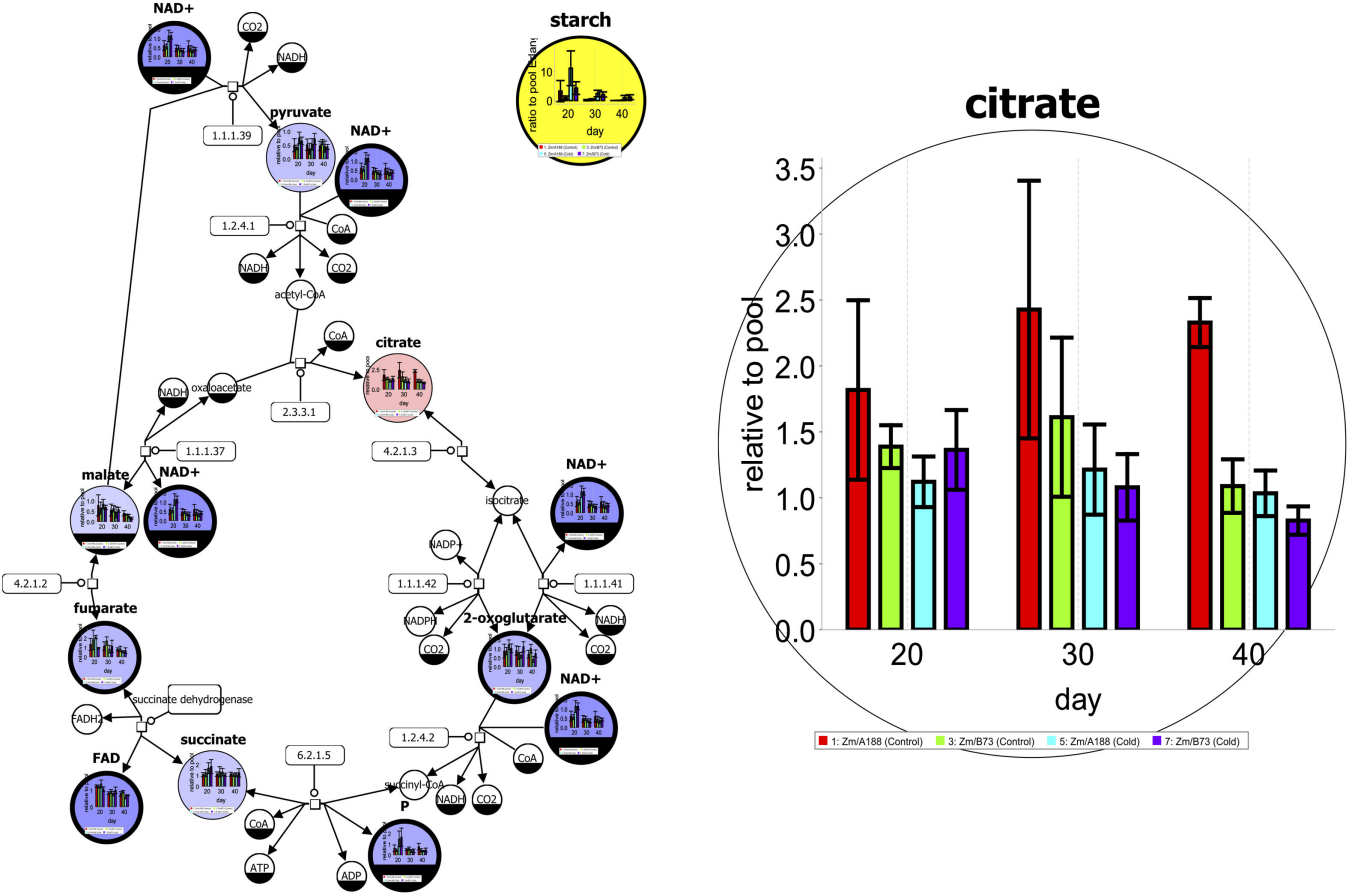
**A metabolic network of the tricarboxylic acid (TCA) cycle in SBGN style with experimental data from Optimas-DW (Colmsee et al., 2012) [produced using Vanted (Junker et al., 2006)]**. More details are given in the text.

Figure 1 shows an exemplary metabolic network in SBGN style (see section Standards for Systems and Synthetic Biology). Time series data for different genotypes and treatments have been mapped on the tricarboxylic acid (TCA) cycle pathway. The left figure not only shows the data using box plots but also a Pearson’s correlation analysis for starch production (yellow). Red- and blue-colored elements have a positive or negative correlation, respectively. For further investigation, single elements can be enlarged to support additional exploration of data as shown on the right side.

### Databases and Repositories for Metabolites and Metabolism

**Figure 2:**
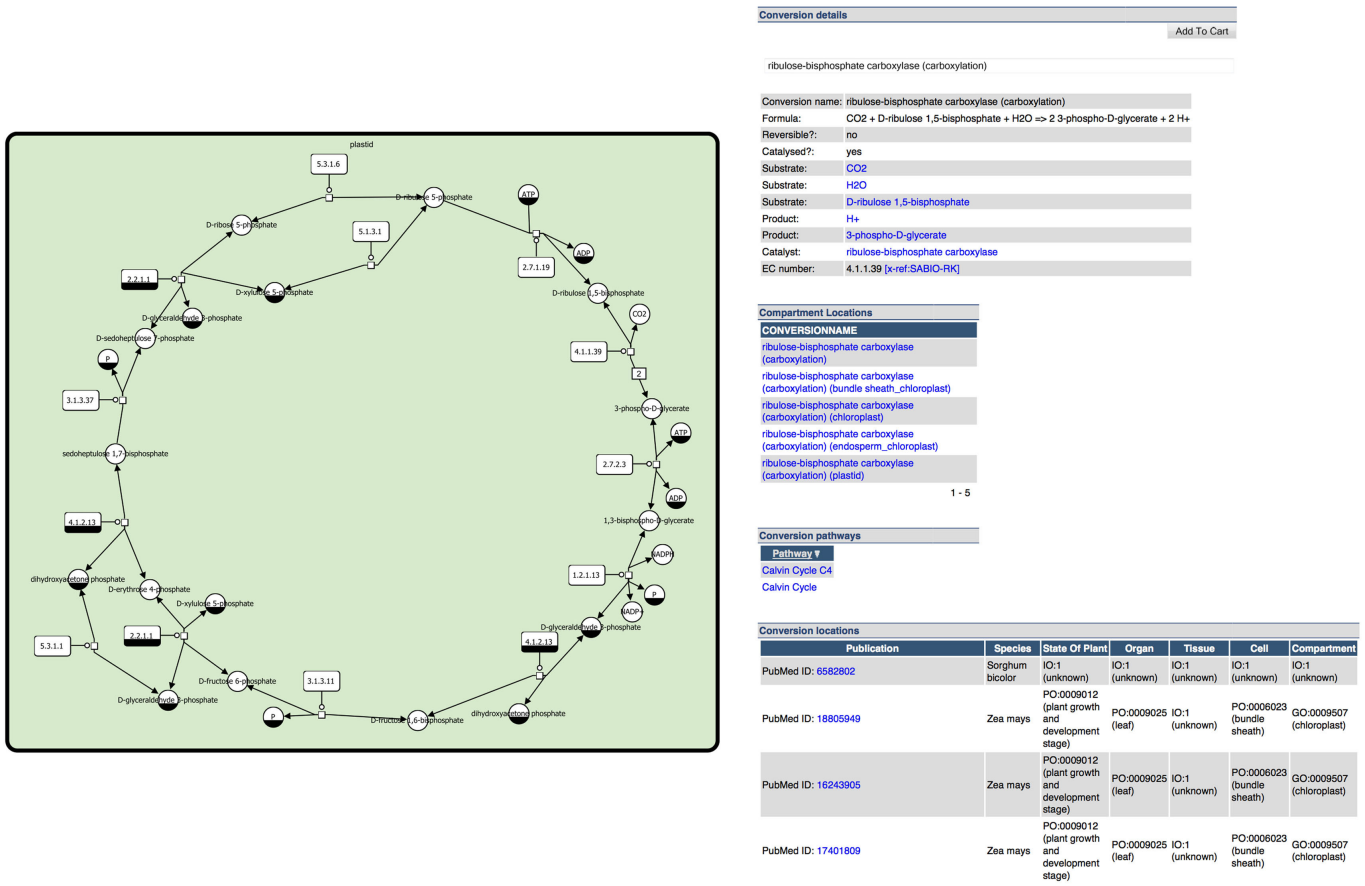
**Example information from the plant metabolic pathway database MetaCrop (Grafahrend-Belau et al., 2008):** (left) clickable image of Calvin cycle represented in SBGN (see section Standards for Systems and Synthetic Biology) and (right) detailed information for a specific reaction of the Calvin cycle.

Given the tools introduced before, metabolite and pathway databases and repositories are valuable resources that can be used to manage, explore, and export knowledge about metabolites and reactions in meaningful ways. They thereby deliver an encyclopedia on metabolic information as well as a base for integration of complex data into metabolic pathways in the context of graphical pathway representations (e.g., data about compound levels, reaction flow, enzyme activity, and gene expression, see Metabolomics Data Analysis and Visualization). Often these databases and repositories also provide or allow building metabolic models which can be analyzed and simulated using mathematical modeling techniques, see Section “Methods for Metabolic Modeling in Plants.” A typical example of information provided by databases is shown in Figure 2.

A range of pathway databases and repositories are currently available; an overview is available from the Pathguide resource (Bader et al., 2006). In Table 3, we provide a summary of important databases and repositories especially for plant research. It should be noted that in addition to general metabolic pathway databases such as Reactome, KEGG (Kyoto Encyclopedia of Genes and Genomes), and PANTHER Pathway, there are also general plant metabolic pathway databases (see Table 3) and several species-specific plant metabolic pathway databases available, for example, for *Arabidopsis* AraCyc (Mueller et al., 2003) and MetNetDB (Yang et al., 2005).

**Table 3:** **A summary of important databases and repositories for plant research**.

Database	Exchange (download) formats	Reference	URL
**Metabolite databases (provide information about metabolites/compounds such as names, chemical structures, molecular weight, occurrence in pathways, EC number, and mass spectrum references)**			
ChEBI	XML, SDF, Tab-delimited	De Matos et al. (2009)	http://www.ebi.ac.uk/chebi/
GMD	MS formats	Kopka et al. (2005)	http://gmd.mpimp-golm.mpg.de/
KEGG compound	Jmol, MDL/MOL, KCF, KegDraw	Kanehisa et al. (2014)	http://www.genome.jp/kegg/compound/
PubChem	XML, SDF, SMILES	Bolton et al. (2008)	http://www.ncbi.nlm.nih.gov/pccompound

**Reaction databases (provide information about reactions and enzymes such as names, reaction diagrams, reaction mechanisms, enzymatic parameters, occurrence in pathways, and links to encoding genes)**			
BRENDA	SBML, Fasta, CVS	Scheer et al. (2010)	http://www.brenda-enzymes.org/
ExPASy-enzyme	–	Gasteiger et al. (2003)	http://enzyme.expasy.org/
KEGG enzyme/KEGG reaction	–	Kanehisa et al. (2014)	http://www.genome.jp/kegg/reaction/
Rhea	BioPAX, Tab-delimited	Morgat et al. (2015)	http://www.ebi.ac.uk/rhea/
Sabio-RK	SBML	Rojas et al. (2007)	http://sabio.villa-bosch.de/

**Pathway databases (provide information about plant-specific metabolic pathways such as names, involved reactions, metabolites and enzymes, and pathway structure)**			
KEGG pathway	KGML, BioPAX	Kanehisa et al. (2014)	http://www.genome.jp/kegg/pathway.html
MetaCrop	SBML, SBGN-ML	Schreiber et al. (2012)	http://metacrop.ipk-gatersleben.de/
MetaCyc	SBML, BioPAX	Krieger et al. (2004), Caspi et al. (2006)	http://metacyc.org/
PANTHER pathway	SBML, BioPAX, SBGN-ML	Mi et al. (2013)	http://www.pantherdb.org/pathway/
PlantCyc	SBML, BioPAX		http://www.plantcyc.org/
Reactome	SBML, BioPAX	Croft et al. (2014)	http://www.reactome.org/

Examples for multispecies plant metabolic databases are:

- *PlantCyc*, which contains curated information on pathways, metabolites, reactions, genes, and enzymes,
- *Arabidopsis Reactome*, which has been extended from the initial representation of *Arabidopsis* pathways to further plant species and contains curated information about pathways, experimental evidence, literature citations, and further pathways imported from KEGG and AraCyc, and
- *MetaCrop*, which focuses on crop plant species with agronomical importance and contains curated information about pathways, metabolites, reactions, enzymes (including kinetic information), location, and developmental stage.

## Current Status of Engineering Synthetic Metabolic Networks

For a long time, the use of biological organisms for the production of chemicals was limited by the repertoire of biosynthetic pathways naturally present in these organisms. Microbial low-molecular-weight metabolic end products have long been used as commodity chemicals for medical applications: for example, antibiotics, such as penicillin, or immunosuppressive drugs, such as cyclosporine, have been discovered in 1929 and 1972, respectively (Drews, 2000). Microbes have also been used to produce chemical compounds via biotransformation, a process in which the compound of interest is produced by the microorganism through enzymatic conversion of an external substrate added to the microbial culture medium, used, for example, for the production of acrylamide by nitrile-assimilating bacteria (Asano et al., 1982).

With the advent of recombinant DNA technology in the early 1990s, it was possible to engineer specific genes in biological organisms, which has significantly reduced the time required for mutagenesis and selection of desirable traits. Genetic engineering made it possible to use heterologous hosts for the production of chemical compounds that are not naturally present in the organism. The emergence of the clustered, regularly interspaced, short palindromic repeat (CRISPR) and related technologies that use targeted genome editing via engineered nucleases are the latest developments to introduce alterations of genome sequences and gene expression, which can be ultimately used to also introduce modifications to existing metabolic pathways and to transfer novel traits in agricultural crops (Shan et al., 2013; Sander and Joung, 2014).

The development of new sophisticated genomic sequencing and other enabling technologies for synthetic biology facilitated the production of naturally present chemicals at levels that made extraction economically feasible, and the field of metabolic engineering began to emerge. Metabolic engineering is defined as the targeted modification of metabolic pathways of biological organisms for metabolite overproduction or the improvement of cellular properties (Lessard, 1996). Since the last decade, significant progress was made to engineer the metabolism of plants to produce specific lipids, secondary metabolites, derivatives of complex natural products, and even vaccines (Mortimer et al., 2012). Many recent studies show that it is often not sufficient to modify existing metabolic pathways, but rather it is required to design metabolic pathways *de novo* from other plants or bacteria. Editing or redesigning existing plant metabolic networks is a challenging task that will benefit from advances in targeted genome modification, tissue-, cell-, and organelle-specific gene expression, the controlled expression of multigene pathways, and improvements in analytical technologies (as described in Analytical Technologies) as well as computational analysis and modeling methods (as described in Metabolomics Data Analysis and Visualization, Databases and Repositories for Metabolites and Metabolism, and Computational Approaches for Metabolic Engineering).

The ultimate goal of synthetic biology is the efficient design of biological systems (Heinemann and Panke, 2006). In this section, we will discuss the current status of engineering synthetic metabolic networks using recent examples for synthetic biology endeavors in plants: engineering of synthetic metabolic networks of plant lipids to provide an alternative and sustainable source of nutrients (see Metabolic Engineering of Plant Lipids to Provide an Alternative and Sustainable Source of Nutrients) and for the production of fuels from renewable resources (see Metabolic Engineering of Plant Lipids for the Production of Fuels from Renewable Resources), and plant secondary metabolites including alkaloids and lignins (see Metabolic Engineering of Plant Secondary Metabolites).

### Metabolic Engineering of Plant Lipids to Provide an Alternative and Sustainable Source of Nutrients

Plant oils are a major component of human diets, comprising as much as 25% of average caloric intake (Broun et al., 1999). However, certain fatty acids such as omega-3 long-chain polyunsaturated fatty acids (ω3 LC-PUFA) are present predominantly in fish and have important functions for human health, as deficiencies in these fatty acids can increase the risk or severity of cardiovascular and inflammatory diseases (Abeywardena and Patten, 2011). Until recently, the chemical composition of plant oils was constrained by the repertoire of naturally present lipid biosynthetic pathways. Novel opportunities have emerged to tailor the composition of plant-derived lipids so that they are optimized with respect to food functionality and human dietary needs. For example, Petrie et al. (2010, 2012) have recently described metabolic engineering of ω3 LC-PUFA in plants: after inserting seven biosynthesis genes of the docosahexaenoic acid (DHA) biosynthesis pathway from microalgae into the genome of *Arabidopsis thaliana*, they were able to obtain ω3 LC-PUFA levels in seeds similar to that observed in bulk fish oil. If applied to oilseed crops such as *Brassica napus*, this technology could potentially form the basis of a plant-based sustainable source to complement the existing marine fish oil supply.

### Metabolic Engineering of Plant Lipids for the Production of Fuels from Renewable Resources

Fossil fuels are the primary source of many industrial products, but reserves are decreasing rapidly and are non-renewable, and their widespread use has contributed to environmental problems arising from increased CO_2_ levels in the atmosphere (Le Quéré et al., 2009). Currently, biologically derived fuels from plant oils represent one of the main strategies to provide renewable and sustainable source material that can potentially substitute fossil fuels in some industrial applications. Among the many proposed solutions, algal biofuels are seen as one of the most promising: algal biomass is less resistant to conversion into simple sugars than plant biomass due to lack of lignin, and there is no issue arising from the food versus feed dilemma as no farmland has to be diverted for the production of biofuels (Daroch et al., 2013). Over the last few years, progress was made in bioethanol production through fermentation from algal feedstock (Kim et al., 2012) as well as biodiesel production from algal oils (Singh and Dhar, 2011). Increasingly, research efforts are focusing to metabolically engineer lipid pathways to increase lipid accumulation without compromising growth (Trentacoste et al., 2013). Although previously *A. thaliana* mutants of lipid catabolism were found to be linked with impaired growth (Graham, 2008), Trentacoste et al. (2013) demonstrated that disrupting lipid catabolism via the knockdown of a multifunctional lipase/phospholipase/acyltransferase in the microalgae *Thalassiosira pseudonana* led to an increased lipid accumulation without compromised algal growth. Further elucidation of lipid metabolism has the potential to lead to new strategies to engineer improved algal strains for their fuel molecules.

### Metabolic Engineering of Plant Secondary Metabolites

Plant secondary metabolites, such as alkaloids, flavonoids, terpenes, and phenylpropanoids (Hill et al., 2014), are considered to be non-essential for normal growth and development but play important roles in plant defense against pathogens and other environmental stresses. Additionally, plant secondary metabolites are of great interest to pharmaceutical industries, as they often have beneficial medicinal effects on humans. For example, many plant alkaloids are currently in medical use, such as atropine derived from the nightshade *Atropa belladonna*, morphine from the opium poppy *Papaver somniferum*, and quinine from the *Cinchona* tree (Roberts and Wink, 1998). Recent progress has been made in the metabolic engineering of morphine, a medicinally important benzylisoquinoline alkaloid: Runguphan et al. (2012) reengineered a codeine *O*-demethylase mutant that selectively demethylates codeine instead of both codeine and thebaine, as is common in the wild-type morphinan biosynthesis pathway. The integration of this highly selective mutant enzyme into commercial poppy plants as part of a future metabolic engineering effort has the potential to increase yields of morphine and codeine.

The phenylpropanoid pathway is conserved in all terrestrial plants and is responsible for the biosynthesis of many compounds that are involved in plant cell wall structure and integrity, water transport, and plant defense. They are required for the biosynthesis of lignins, aromatic natural polymers in secondary cell walls derived from the oxidative polymerization of monolignols. Decreasing or altering lignin structure provides enhanced cell wall digestibility and can greatly increase the utilization of lignin itself or cell wall polysaccharides. Due to the importance of lignin in agriculture and industry, the genes participating in lignin biosynthesis have been identified and modified in many plant species including switchgrass (Fu et al., 2011), *A. thaliana* (Gallego-Giraldo et al., 2011), and sugarcane (Jung et al., 2012). In a recent study, Zhang et al. (2012) were able to manipulate lignification in *A. thaliana* without compromising plant growth by introducing an artificial enzyme that esterifies the para-hydroxyl of phenols. The modified 4-*O*-methyl lignin monomers deprive the products of participation in oxidative dehydrogenation, leading to a decreased level of available monolignols for lignin polymerization and thus to depressed lignin biosynthesis. Further metabolic engineering efforts are currently underway to integrate this artificial enzyme into poplar with the potential to manipulate lignin levels.

## Computational Approaches for Metabolic Engineering

A variety of different methods and approaches to collect experimental data can be used to quantify metabolites and other components of regulatory networks in plants, such as metabolomics (see Analytical Technologies). Computational modeling is an important tool for metabolic engineering as it facilitates the integration and analysis of experimental datasets to quantify metabolic fluxes and model metabolic networks. Section “Methods for Metabolic Modeling in Plants” presents a brief overview of modeling approaches. There are many tools and databases available for computational modeling and therefore a standardized exchange of models is highly relevant; Section “Standards for Systems and Synthetic Biology” provides an introduction to major standards in systems in synthetic biology.

### Methods for Metabolic Modeling in Plants

Several approaches have been developed to qualitatively and quantitatively model and simulate metabolic systems *in silico*. This ranges from topological analysis of network models (which looks at the interconnections between metabolites) to stoichiometric models (where constraints can be applied to define the potential metabolic flux state space or which can be analyzed using Petri-nets) to detailed kinetic models (which model changes of metabolite concentration over time). A current review (Baghalian et al., 2014) discusses the different modeling approaches, modeling software, and metabolic models of several plants in detail. A particular challenge in plants compared to prokaryotic cells is the number of different compartments, which needs to be considered in metabolic models. In addition, plants have a greater complexity of metabolic pathways and especially a large number of special pathways for secondary metabolites.

**Figure 3:**
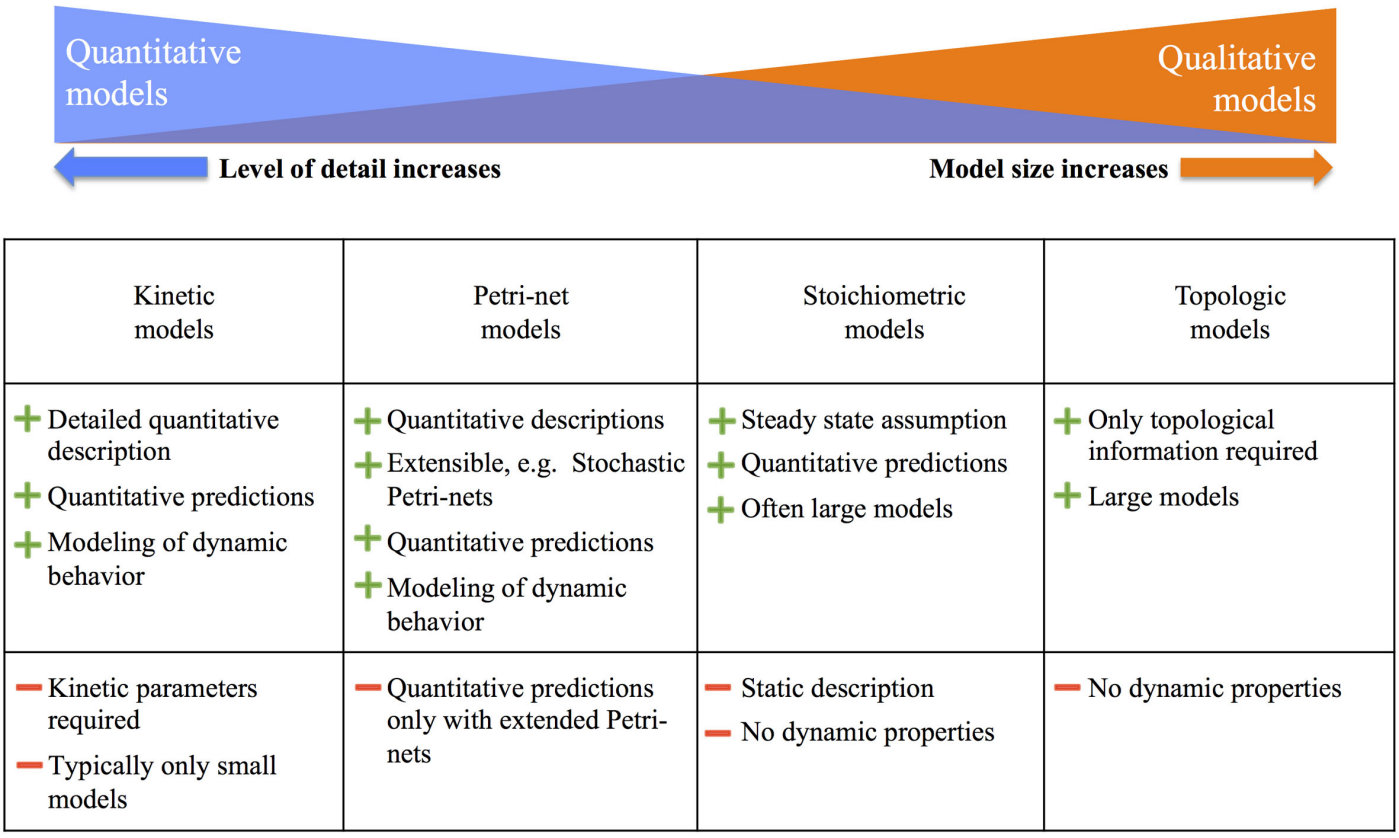
**Overview of metabolic modeling approaches and their advantages and disadvantages, adapted from Hartmann and Schreiber (2014)**. More details are given in the text.

For an overview, Figure 3 summarizes the major modeling approaches and their advantages and disadvantages. The most detailed are kinetic models, which allow for a comprehensive quantitative description and prediction of metabolic fluxes. However, in plants, these models are common only in the size of 10–20 reactions. As we move further to the right in Figure 3, the model size increases, but the level of detailed descriptions and predictions decreases. As the other extreme, topological models allow covering the complete metabolism in plants, but predictions are restricted to qualitative information such as reachability of metabolites.

As detailed in (Baghalian et al., 2014), plant-specific computational models of metabolism can be used for different purposes such as predicting the behavior of the metabolism under different conditions, analyzing the effect of mutations, and investigating the effect of changes due to manipulation of the metabolic system, for example, via the introduction of new metabolic pathways. Computational models usually allow investigating effects much faster and cheaper than by running wet laboratory experiments (Rohwer, 2012). In addition, using a computational model can often generate a set of alternative strategies (Copeland et al., 2012). Finally, computational models allow integrating additional data such as transcriptomics and proteomics data sets, which together with bioinformatics approaches can support a better understanding of metabolic behavior in plants (Töpfer et al., 2012, 2013).

### Standards for Systems and Synthetic Biology

This section presents a short introduction to major standards in systems and synthetic biology related to software infrastructure (see also Table 4). Software infrastructure plays an important role in systems biology research (Kitano, 2002), in particular in supporting standardized exchange of information between different tools and databases. The major standards are Systems Biology Markup Language (SBML), CellML, Systems Biology Graphical Notation (SBGN), and Synthetic Biology Open Language (SBOL), Language (SBOL), see (Schreiber et al., 2015) for detailed specifications.

**Table 4:** **Overview of major standards in systems and synthetic biology**.

Name	Reference	URL
SBML	Hucka et al. (2003)	http://sbml.org/
CellML	Cuellar et al. (2003)	http://www.cellml.org/
SBGN	Le Novère et al. (2009)	http://sbgn.org/
SBOL	Galdzicki et al. (2014)	http://sbolstandard.org/

#### Systems Biology Markup Language

Systems Biology Markup Language is a machine-readable format for representation and exchange of computational models in systems biology. It can represent models of metabolism, signal transduction, and gene regulation. The main goals of SBML are (1) sharing and publication of models, (2) reusability of models, and (3) surviving of models beyond the lifetime of the software used to create them. The SBML web page currently lists more than 270 software applications that support SBML, and thousands of SBML-encoded models are available from public repositories such as BioModels including Path2Models (Büchel et al., 2013; Chelliah et al., 2013).

An SBML model consists of hierarchical lists of conceptual elements: (1) species (biological entities taking part in reactions), (2) compartments (physical containers for species), and (3) reactions (transformation, transport, or binding processes occurring over time). For the analysis and simulation of a model, more properties need to be defined such as stoichiometries, rate laws, local and global parameters, as well as units on quantities. A formal description of SBML can be found in the detailed specification (Hucka et al., 2015).

#### CellML

CellML is a machine-readable format for representation, publication, and sharing of mathematical models of cellular function. In comparison to SBML, the focus of CellML is on the representation of a variety of models such as models of biological pathways, electrophysiological models, and mechanical models. The CellML web page lists a couple of software tools which support the CellML format and the development of models. The CellML Model Repository (Lloyd et al., 2008) contains several hundred models including a subrepository providing SVPs (Standard Virtual Biological Parts) for the composition of synthetic biology models (Cooling et al., 2010).

A CellML model description consists of components and lists of connections between the components. A component contains at least one variable and mathematical equations describing its behavior. Connections are mappings of variables between components enabling information exchange between them. Components and connections can be imported from an existing model, CellML allows reusing of parts of other models. A detailed description of CellML can be found in the specification (Cuellar et al., 2006).

#### Systems Biology Graphical Notation

Systems Biology Graphical Notation is a standard for the graphical representation of processes and networks studied in systems biology. Three SBGN languages (Process Description, PD; Entity Relationship, ER; and Activity Flow, AF) allow for the representation of different aspects of biological systems at different levels of detail as SBGN maps, thus providing corresponding views on the underlying biological system. The PD language (Moodie et al., 2015) describes biological entities and processes between these entities, the ER language (Sorokin et al., 2015) focuses on interactions between biological entities, and the AF language (Mi et al., 2015) depicts information flow between biological activities. An example of an SBGN PD map is shown in Figure 1.

The standardization of graphical representations helps to exchange biological knowledge more efficiently and accurately between different research communities, industry, and other players in systems biology. Several databases already provide maps in SBGN, e.g., BioModels Database including Path2Models (Büchel et al., 2013; Chelliah et al., 2013), MetaCrop (Schreiber et al., 2012), PANTHER Pathway (Mi et al., 2013), Reactome (Croft et al., 2014), and RIMAS (Junker et al., 2010). The SBGN web page lists more than 20 software tools that support creating, editing, and viewing of SBGN maps, some of these tools allow to visualize SBML models in SBGN PD.

#### Synthetic Biology Open Language

Synthetic Biology Open Language is a data format for sharing and exchanging synthetic biology designs. It allows synthetic biologists to provide an unambiguous description of a design in a hierarchical and fully annotated form with the goal to improve designing, building, testing, and dissemination of synthetic biology designs. For the visualization of synthetic biology designs in SBOL, SBOL Visual (Synthetic Biology Open Language Visual) has been developed. It is a graphical notation allowing depiction of the structure of a design using glyphs to specify genetic parts, devices, modules, and systems.

The SBOL web page currently lists more than 20 software applications supporting SBOL and SBOL Visual. Some of these applications allow the generation of SBML models from synthetic biology designs in SBOL (Roehner et al., 2015) as well as the creation of synthetic biology designs in SBOL by automatically generating DNA sequences from annotated SBML and CellML models (Misirli et al., 2011). More detailed information about SBOL and SBOL Visual can be found in the specifications (Quinn et al., 2013; Bartley et al., 2015).

## Conclusion

The total number of metabolites in the plant kingdom is estimated to be between 100,000 and 200,000 and can be highly variable depending on the physiological and environmental conditions as well as the genetic background of the plant (Hill et al., 2013a,b). Such great metabolic diversity holds great promise for expanding our repertoire of known beneficial plant compounds, as many metabolic pathways and regulatory mechanisms are still awaiting discovery. Reaching significant benchmarks toward attaining these goals will be possible with better analytical tools. More accurate representations of metabolite identities and quantities will require analytical instruments and improved techniques for sample extraction and data analysis. The engineering of synthetic metabolic networks of plants will require further advances in targeted genome modification such as the application of the CRISPR/Cas system, as well as tissue-, cell-, and organelle-specific gene expression, and the controlled expression of multigene pathways. The development of methods for measuring metabolic flux directly, the quantification of metabolites in individual plant compartments, and the analysis of metabolites and activities between compartments *in vivo* will be very important next steps to further enhance the predictive capabilities of existing metabolic models. Continuous development of more user-friendly software, databases, languages, and computer models that incorporate and interpret complex information will be crucial to handle the acquired data and to aid interpretation in a biological context. We are just at the beginning of a new area of synthetic biology in plants based on metabolomics and metabolic modeling.

## Conflict of Interest Statement

The authors declare that the research was conducted in the absence of any commercial or financial relationships that could be construed as a potential conflict of interest.
